# Challenges and executive requirements of advanced health system governance based on general health policies in Iran: qualitative research

**DOI:** 10.1186/s12913-024-11887-z

**Published:** 2024-11-30

**Authors:** Masoud Aboulhallaj, Seyed Masood Mousavi, Mehdi Jafari, Abbas Vosoogh-Moghaddam, Sajjad Bahariniya, Shabnam Ghasemyani, Sedighe Sadat Tabatabaei Far

**Affiliations:** 1 Healthcare Services Management Iran Ministry of Health and Medical Education, Tehran, Iran; 2 Health Policy and Management Research Center, Department of Health Management & Economics, School of Public Health, Shahid Sadoughi University of Medical Sciences, Yazd, Iran; 3 Department of Healthcare Services Management, School of Health Management & Information Sciences, Iran University of Medical Sciences, Tehran, Iran; 4 Governance and Health Training and Research Department, National Institute for Health Research, Tehran University of Medical Sciences (TUMS), Tehran, Iran; 5 PhD Candidate of Healthcare Services Management, Student Research Committee, School of Health Management and Information Sciences, Shiraz University of Medical Sciences, Shiraz, Iran

**Keywords:** Health system, Policies, Health policy, Governance, Stewardship

## Abstract

**Objective:**

The optimal management of the health system depends on its functions. The functional issues of Iran’s health system are organized in a scattered manner and it has many challenges, especially in the field of stewardship. In order to achieve sustainable health-oriented development, bold and smart policy reforms in the main components of the health system are essential. The purpose of this study was to identify challenges and institutional-operational requirements in order to promote stewardship based on the general health policies of the country.

**Methods:**

This study was conducted qualitatively with a framework analysis approach on policy makers and health experts. Purposive sampling was done with maximum diversity. To achieve a comprehensive view, snowball sampling was completed, resulting in the participation of 47 individuals in this study. A semi-structured interview was used to collect data. The analysis was done in MAXQDA software.

**Results:**

Challenges and requirements for the implementation of General Health Policy tasks in promoting the function of stewardship, according to the World Health Organization model, in 3 fields of vital evidence production and policymaking (4 main topics and 20 sub-topics), internal governance (6 main topics and 23 subtopics) and Inter-Sectoral Leadership (2 main topics and 6 subtopics) were categorized. The most important challenges in the field of stewardship included fragmentation, the lack of an integrated information system and coherent and coordinated evidence production institutions, and weakness in the supervisory dimension due to the fatness of the structure, conflict of interests, and lack of transparency in responsibilities.

**Conclusion:**

It seems that the creation of comprehensive governance structures in the form of a network in active interaction with the Secretariat of the Supreme Council of Health and Food Safety, the development of the information system, the coordination of vital evidence production institutions, capacity building to strengthen intra-sectoral governance and inter-sectoral leadership and create a suitable platform be effective for public participation.

**Supplementary Information:**

The online version contains supplementary material available at 10.1186/s12913-024-11887-z.

## Background

Optimally managing a country’s health system depends significantly on its specific functions and components [1]. The interrelation of these functions and components is a key issue [2]. In this context, the World Health Organization’s approach has significantly strengthened health systems [1, 2]. Determining the components of the mentioned approach alone does not lead to the improvement of the performance of the health system, and policymakers should look at it with systemic thinking and consider the multiple relationships and opposition of these components with each other as a system [3, 4]. Strengthening health systems to achieve the goals of sustainable development and Universal Health Coverage requires evidence-based policy intervention [3].

Governance is a set of legal mechanisms and regulations established by the parliament and government and are finally implemented by the government body. Governance is the creation of a system for better management of the government, organization, or social group and is ultimately regulated with the aim of accountability and responsibility [4].

Health policy, as one of the main dimensions of social policy, is a set of policy decisions and programs that the government adopts to expand public health and improve the capabilities of citizens to reduce [5–7]. To achieve sustainable development of health, bold and clever policy reforms in the main components of the health system, that is, governance, provision of health services, and financing, are necessary [8]. The changing needs of societies that the health system must respond to make the reform of this system inevitable in all countries worldwide [9]. Countries have implemented a wide range of reforms in financing and providing health services to move towards Universal Health Coverage [10].

Health systems are becoming increasingly complex, and the Ministry of Health must coordinate with a set of factors and be accountable for guiding health systems [11]. The ministries in charge of health have a unique role in health care because of their exclusive powers according to the constitution or approval by the government on this matter. These ministries provide the direction and perspective of the health system and play a fundamental role in the legislation and management of basic public health functions. At the country level, the Ministry of Health should coordinate and apply its governance roles with other organizational actors [11, 12].

Today, a concept called “Good Governance in the health system” is proposed, which is key to the puzzle of development in today’s complex world and New Public Management. Good Governance is a type of decision-making and implementation that includes the features of justice, rule of law, participation, consensus, transparency, effectiveness and efficiency, responsibility, and accountability. Mismanagement, poor Intersectoral coordination, lack of transparency, and accountability have caused the overall health policies in Iran to not achieve the desired results [13–15].

According to Article 29 of the Constitution and the structure of Iran’s political economy, the government is considered the main player in health policy [16]. There are three main hierarchical leadership levels in the health system of Iran, each with its own specific leadership and governance functions. There are two multi sectoral bodies designated for health affairs. By law, MOHME acts as the steward of the health system, makes decisions about various functions including public health, curative affairs, medical research and education, food and drug, among others, and reaches out to the entire population for healthcare through currently 67 medical universities across 31 provinces in Iran Healthcare services are delivered through three main ways in Iran: the public-government system, the private sector, and non-governmental/charity organizations. The key reform at the heart of this progress has been the establishment of an extensive PHC Network since 1985 [8].

Health financing in Iran is a mixed system, predominantly funded through the public budget, health insurance funds and OOP payments. IHIO currently affiliated with the MOHME, along with SSO and MOCWSL are the main providers of public health insurance in Iran. Health Workforce to overcome serious shortages in human resources for health (HRH), the medical education was integrated into healthcare services to create the MOHME in 1985. Although it is not still optimal, particularly in some medical specialties within remote and deprived areas, the strategy has been successful to produce the ever-increasing number of required HRH across the country. In 2015, the MOHME created the comprehensive roadmap of the required HRH by 2025, which is being used to regulate intake and distribution of healthcare personnel across the nation. It is also worth mentioning that an evolutionary and innovative plan for the medical education using forecasting approach [8].

Despite the importance of integration, the functional issues of the health system in Iran are now fragmented, and there is interference in authority, especially in the field of supervision [5]. In other words, many organizations and institutions play different and sometimes conflicting roles and usually cause changes in goals and results [6]. Regarding the other important functions of the health system, there are also shortcomings such as the challenge of access, continuity, comprehensiveness and quality of services, effectiveness and efficiency of services, the high share of direct payment from patients’ pockets, justice in benefiting from medical services and justice in paying health costs [17–22], We can also mention the challenges in the country’s pharmaceutical system, the leveling of health services and the referral system, the development and application of clinical guidelines, health service tariffs, and the relative success of the hospital self-management plan [8–10].

The health system of Iran has undergone a multitude of structural and organizational reforms in recent decades [23]; however, regrettably, the structural modifications within the Ministry of Health in recent years have predominantly been predicated upon a prevailing perspective that emphasizes the augmentation of responsibilities and obligations aimed at garnering financial resources, enhancing political legitimacy, and sustaining and advancing the status of certain administrators. This situation has culminated in a substantial burden of educational, research, and therapeutic responsibilities within the Ministry of Health and Medical Education, thereby compromising the oversight of Iran’s health system [24].

The prevailing legislative frameworks have underscored the significance of health and equitable access to healthcare services as inherent rights afforded to all citizens of Iran [25, 26]. In this context, overarching health policies were promulgated in 2013 with the objective of attaining the preeminent status of health indicators within the nations of the Southwest Asian region, through the implementation of a holistic approach to health and the promotion of well-being among the populace in all legislative, regulatory, and executive policies. In the seventh paragraph of these policies, the three main functions of the health system are mentioned, so that the separation of the duties of responsibility, financial provision, and provision of services in the field of health with the aim of accountability, justice, and providing optimal medical services to the people, taking into account the role of responsibility of the health system, including executive policies, strategic planning, evaluation, and monitoring by MOHME; This research was conducted to identify Challenges and Executive Requirements of Advanced Health System Governance Based on General Health Policies.

## Method

This qualitative study which aimed to investigate the challenges of implementing S7GHP. Both purposive and snowball sampling methods were used to recruit the participants. To meet the maximum diversity, the research team tried to consider different individuals by their experience, expertise, and knowledge about the macrostructure of The Health Sector, Stewardship, Financing, and Service Provision in Iran. Considering that the Ministry of Health has a more important role in formulating and implementing the mentioned policies, more participants from this ministry were selected for the interview. Initially, individuals were selected directly based on the familiarity of the research team members, but in the later stages, the interviewees were asked to introduce appropriate individuals for next interviews. Sampling was continued until data saturation was achieved. Also, two interviews with duplicate data were considered to ensure data saturation. Forty-seven participants Appendix 1 shows more details of participants. Based on the ethics principles, participants were free to leave the study at any stage. It should be noted that the interview guide file is attached (Appendix 2).

Semi-structured interviews were done by using an interview guide in order to improve the management of the interview process. The interview guide included a number of open-ended questions developed based on the S7GHP. During the first two interviews, the problems and shortcomings of the interview guide were examined and then the questions were corrected and revised for more clarity. As a whole, the interview guide consists of three main sections including 1) background of macro-structure reforms of the health system, 2) S7GHP and their components, 3) challenges of main components of S7GHP, and 4) applied recommendations for the implementation of S7GHP. To have an in-depth interview, some probes were considered during interview sessions. Interviews were held in a quiet room with only the interviewer and the interviewee in attendance. In addition to audio recording, notes were taken during the interviews. The recorded files were transcribed in text and after adapting and completing relevant notes and documents by the one of the authors (M.M), the collected data were considered for analysis. To keep the participants anonymous, identification numbers were used instead of their names in the documents. To meet the critical reflexivity, the interviewer fully described his academic background at the beginning of each session Standards for Reporting Qualitative Research (SRQR) [27] and the consolidated criteria for reporting qualitative research (COREQ) [28] were applied throughout the study to promote the quality of reporting.

The framework analysis approach was considered to investigate the opinions of participants [29]. This study applied a combination of the two frameworks of the World Health Organization (WHO2000) and the components of the health system (Building Block 2007) [30]. However, because some of the extracted themes could not be used in the adopted framework, the research team decided to use thematic analysis in some findings, in addition to the framework analysis, to be able to provide most of the valuable findings. This step was done in parallel with the data collection process. The two authors (SM.M, S.B) participated in the process independently and read the written texts frequently. Based on the framework approach, the meaning units were identified first, and then by condensing them, the codes were revealed. In the next step, the codes were reviewed and merged to organize the sub-themes. Eventually, the research team, after holding several discussion sessions, allocated the sub-themes to pre-defined themes. Specifically, the authors involved in the data analysis process had different scientific and professional backgrounds that could reduce potential bias.

To secure the rigor and trustworthiness of qualitative findings, the research team considered a number of methods to meet the dependability, transferability, conformability, credibility, and authenticity [31]. Several members of the research team with different scientific and professional backgrounds were involved in data analysis to cover the dependability criterion. Also, to enhance the transferability level of findings, purposive sampling with maximum diversity was applied. Member checking approach by participants was used to meet the conformability. Furthermore, data triangulation and prolonged involvement were considered to promote credibility. Finally, to enhance the authenticity, the research team tried to use citations from different participants.

## Result

Paragraph 7 of the general policies of the health system targets the three functions of stewardship, financing, and providing services. We undertook an analysis of the obstacles and prerequisites associated with the enactment of the stewardship function, drawing insights from the perspectives of the interview participants.

### The function of stewardship

According to the model of the World Health Organization in 2000, stewardship includes three fields of evidence informed policy making, internal governance and inter-sectoral leadership. In general, in the field of vital evidence and policy making, four main topics and 20 sub-topics were classified. In the field of intra-sectoral governance, six main topics and 23 sub-topics, and the field of inter-sectoral leadership, two main topics and six sub-topics were classified.

### Evidence informed policy making

This field included four main issues (inappropriate structure, uncoordinated processes of producing evidence, improper decision-making and management style and insufficient resources). In this regard, subtopics were extracted for each of the challenges.

#### Inappropriate structure

Regarding this challenge, some of the interviewees pointed to “defects in adhering to the plan and not being forward-looking of the ministry”. One of the managers mentioned that “An urgent letter comes from the ministry to the universities which destroys all the strategic plans, and then becomes the priority.” (P12) emphasized the necessity of a systematic approach and the priority of active plans over passive ones.

On the one hand, from the point of view of several experts, due to reasons such as “lack of a powerful trustee” and “dispersion of evidence production institutions,” the system of producing evidence in the health sector is seriously flawed.

A group of interviewees also referred to the issue of “non-integrated stewardship in specialized departments” due to the complexity of the health field and acknowledged that this conflict of policies caused disruption in the implementation process and inconsistency in strategic planning to fulfill the provisions of paragraph 1-7.

The next challenge mentioned was the “weakness of the institutional position of the Academy of Medical Sciences” so that according to paragraph 1-7 of the communicated policies, some words such as executive policy and strategic planning had different meanings.

Therefore, the suggestion of “Strengthening the role of the Academy of Medical Sciences” was proposed for professionalism and uniformity in the application of the broad terms of the health system. One of the interviewees believed that each of the words in addition to the correct scientific translation should be used according to the conditions of the country. In line with this challenge, one of the managers pointed out the most important solution for this issue is “creating an information and evidence management center under the sub-category of the deputy director of policy, planning and supervision” or “establishing Iran’s health statistics, information and evidence organization.”

#### Uncoordinated processes of producing evidence

In the correct definition of the policy options for paragraph 7, general policies were named “different interpretations of the broad terms of policy making in the health system.” According to the interpretation of one of the interviewees, one of the main goals of the design of this section was to strengthen the functions of the health system by reducing the border between the health system and the health sector. One of the reasons for this challenge is the “weakness of ideological discussions” in policymaking. However, from another point of view, some of the interviewees considered “politicization” as one of the problems of the evidence structure. In other words, they believed that many policy decisions are made passively without considering long-term perspectives. Therefore, several experts pointed to the “lack of a network of policymaking experts” as one of the challenges. In confirmation of this issue, some believed that during the years when the health policy council was operating in the ministry, it had valuable outputs, and with the dissolution of this institution, due to various structural, political, and management reasons, a potential capacity in this matter was lost.

On the other hand, a group of academic experts pointed to the “ambiguity in the prioritization of the written policies” of the ministry in the implementation stage and admitted that, unfortunately, the evaluation system is not taken seriously for the same policies that are being implemented. Another group of interviewees also mentioned the issue of “inefficiency in the performance management process” as one of the challenges of the implementation of paragraph 7. Also, “the lack of strong connection between the academic field and implementation” was mentioned as another challenge because many problems in the executive field come from the lack of a clear process between the evidence produced in universities and its use in decisions. With all these descriptions, one of the directors of the Ministry of Health proposed “the formation of a coherent and coordinated network consisting of executive policymakers and university academic professors” to reduce the gap between policy and practice as an implementation solution. One of the managers also introduced “the integration of the country’s health system through the coordination of institutions based on the context, content and service process with the responsibility and centrality of the Ministry of Health” among the proposed requirements.

#### Improper decision-making and management style

The health system has many challenges for effective decision-making due to the “insufficient use of tested evidence” in the policy-making style. One of the participants has emphasized the necessity of using validated evidence by citing the quote “The wrong view of managers who say that everything will be fixed by changing the structure or that research is for long-term work and is not necessary for daily research work” (P6). But a group of experts pointed to the issue of the “short decision-making life of policymakers and managers” as one of the most important challenges: “Political and management changes are so fast that even if a manager wants to institutionalize a policy, his management life is not enough” P14). “Pressing the levers of power to make policies like some MPs” was mentioned as one of the emphasized sub-topics for this challenge. One of the professors of the university comments on this:

*“In our country, policies are not based on evidence and collective wisdom, and I think we only give the slogan of evidence-based policy-making. In practice, our researchers do a lot of work, and then it goes to the main decision-makers; external control levers change the way they make decisions. The reason, in my opinion, is the weak institutional position of evidence production centers in the country” (P47).*

Among the mentioned challenges, the policy-making system has suffered a “separation between policy-making and strategic planning” and the designed programs are not policy-oriented. However, for this important challenge, “designing a comprehensive system of macro-health policymaking or revitalizing the former health policymaking council” by using expert policy experts with non-political positions is suggested.

### Internal governance

The findings of this field include six main issues: the weakness of governance in implementation, the weakness of the role of regulatory institutions, too much tenure of the headquarters, the incorrect architecture of the structure of the headquarters and universities, Contradictory Laws with General Health Policies and weakness in the system of appointing and dismissing managers were classified.

#### Weakness of governance in implementation

Almost the majority of the interviewees believed that the main challenges of reforming the macrostructure of the health system are “lack of serious commitment and determination” and “ambiguity in the executive guarantee of paragraph 7 of general health policies” and “severe conflict of interests at different institutional levels”.

Therefore, although the existence of a conflict of interests cannot be considered a serious reason for corruption or bad behavior, and these two should not be considered as one and their relationship is not independent and direct, they are very much related due to the increased possibility of error. In this regard, the less the integration of policymaking and stewardship in the headquarters, the more the above-mentioned cases will occur.

Also, several managers have considered “factional political competition at the decision-making levels in different layers of the health system” as the main cause of this phenomenon, for example, one of the former managers in the secretariat of the Health and Food Security Council stated that *“Unfortunately, in the 18 years that I have been working in this ministry, the most important challenge is the conflict of interests among the decision-makers at different levels of the Council of Expediency, the Parliament, the Board of Ministers, and the Ministry of Health. ٍEven though health is beyond politics and factional rivalries, there is a conflict of interest at the decision-making levels” (P29).*

On the other hand, some believed that despite the formation of a specialized working group in the compilation of “the implementation of the framework for the establishment of general health policies” in 2013 and the design of the structure for the implementation of the communicated policies, it was not successful in practice and somehow the path of implementation and accountability was sidelined. The most important suggestion was “drafting a specific policy legal framework” by managers and policymakers in the three powers. The requirement of this framework should also be specific laws regarding information transparency and reducing conflict of interest in the health system.

#### The weakness of the role of regulatory institutions

Among the institutional challenges, we can mention “the passive role of the Boards of Trustees of Headquarters and Universities”, which currently do not perform their role properly due to a lack of independence. In this regard, some have called this institution a “formal structure for general and routine decisions” (P30). A group also mentioned the weakness of the supervisory role as a result of “insufficient independence of universities in decision-making” and pointed out that the need for this issue is to change the composition of the board of trustees or to change the regulations. Several professors of the university considered “fragmentation in the regulatory system” as the cause of this problem, that the regulatory processes of these institutions are not aligned and not only do not cause synergy but also cause disorder in the macrostructure.

Some added the issue of “weak supervision of the private sector” to other supervisory challenges (P11, P18). This group believed that despite the existence of a clear law for the organization of the medical system, his institution does not perform its duty well. Some of the interviewees also mentioned the “ambiguity in the role of external institutions’ supervision” as one of the challenges.

Among the proposed requirements, “Strengthening the capacity of the secretariat of the Board of Trustees and the use of control levers, especially budget and financial control tools” and “Approving and correcting the Law of Independence of Universities” were mentioned. Of course, currently the law on permanent orders for the development of the country implicitly refers to this issue. But unfortunately, there is no complete independence in practice.

Among the other proposed requirements were “compilation of governance guidelines, issuance of licenses, certification, and accreditation”, “establishing a deputyship for policy, planning and supervision”, and “setting up an independent supervisory unit at the province and city level”. However, currently, there is no comprehensive program and centralized authority for this issue.

#### Too much tenure of the headquarters

Uncertainty in the limits and size of the government in governance and tenure duties is an issue that has been mentioned in various legal cases. Due to the integration of medical education in the field of service delivery, the workload of tenure in the ministry has increased significantly, and this issue was raised against the provisions of paragraph 7 of the general policies. In this regard, one of the managers pointed out that “the units of the ministry were unanimous in the small and assignable issues that involved themselves” (P8). Several experts also pointed out “excessive dependence of environmental units on headquarters due to conflict of interests” among the challenges related to functional areas in the health system.

Several of the interviewees emphasized the “non-separation of functions” and the review of the existing job descriptions in the same level and upstream units. One of the members of the Secretariat of the Supreme Council of Health Insurance referred to the “scattering of powers”, especially in the field of financing; he pointed out the necessity of combining these powers with the aim of strengthening the governance and supervision system.

*“…. Now some powers are in sub-category structures: Tariff authority is in a corner of the vice president of treatment, and hospital management authority is also more central. The authority to enter new technology is in different places and very scattered, and it is only concentrated and at the disposal of medicine. It is the vice-chancellor of medicine or the authority to issue a license given to the medical system organization” (P11).*

Among the proposed requirements were “reducing the density of executive tasks of the headquarters and delegating authority to peripheral units” and “organizing universities of medical sciences in the form of ten macro-regions and delegating headquarters’ powers to macro-regions and universities”.

#### Incorrect architecture of the structure of headquarters and universities

Some believed that “in the current structure, many organizations play different and sometimes conflicting roles” (P8, P28). In other words, “political discontinuity and lack of integrity in stewardship” was one of the most important challenges. The experts of the Ministry of Health believed that “parallel work and incorrect definition of roles” lead to a decrease in the level of accountability, as well as an increase in conflicts of interest and organizational corruption.

Considering that the policymaker in paragraph 7 of the health policy seeks to strengthen the stewardship role of the Ministry of Health in the macro-decisions of the health system and, on the other hand, considering that universities of medical sciences are defined as the representative of the Ministry in the affairs of the provinces, “designing a miniature and mirror structure for the headquarters and universities” was one of the issues raised around the structural challenges of the health system.

The miniature structure causes the excessive involvement of the ministry in the affairs of the universities, the multiplicity of executive units in the structure of the headquarters and universities, and consequently the reduction of the chain of accountability to the upstream and downstream institutions. However, from another angle, the Ministry of Health is now considered a part of complex organizations, which has the challenge of “not complying with the principles of horizontal, vertical and geographic separation” due to the multiplicity of scattered executive units and the lack of adherence to simple and agile organizational architecture.

Therefore, some experts believed that the current structure of the ministry schematically lacked the implementation of policy, planning and evaluation potential. Thus, “Revitalization of provincial health organizations”, “Creation of independent and semi-independent units” and finally “Reduction of the General Administration in each deputy to a maximum of three to four General Administrations” were proposed because, currently, the structure of the Ministry of Health is executive and interactions and authorities have not been properly defined and distinguished.

#### Contradictory laws with general health policies

One of the important challenges is “invalid and cumbersome laws and regulations in the health system”. In this regard, many of the interviewees pointed out that along with paragraph 4-7 of the general health policies, “legislative approval in line with the implementation of paragraph 7 by the regulatory institutions” is necessary to implement and resolve possible contradictions. Some also referred to “legal chaos of the health system” besides the ambiguity in the position of general policies to evaluate the performance of the provisions of paragraph 7. They pointed out the obvious contradiction of some cases in the documents and laws of the upstream. In this regard, the “Revision of Invalid Laws and Regulations of the Health System” plan was approved in June 2019 with the aim of clarifying, deregulating, updating and making the laws more effective, and somehow, it prepared the ground for this requirement. One of the topics mentioned by the experts was the “low enforcement guarantee of upstream laws”. Therefore, if this is not an upstream law with enforcement guarantees, it will go to the archives. Two of the managers mentioned the issue of “Revision of the Organization Law of the Ministry of Health” as a requirement for the implementation of policy paragraph 7. The approval of laws such as “transparency in the health system” and “conflict of interest in the health system” can be the proposed requirements. In this regard, one of the experts expressed the following opinion: *“One of the reasons for the obvious conflict of interests in the health system is the lack of transparency in various elements. As long as we don’t have the appropriate legal infrastructure for this issue, the same thing will continue to happen” (P38).*

#### Weakness in the system of appointing and dismissing managers

One of the most pressing challenges in the health system is the “weakness in the regulations for the appointment of managers” because management stability is necessary for policy stability, according to some of the interviewees. Also, one of the other challenges is the “non-separation of technical and political positions” in the management body of the ministry, for the issue of making decisions about improving the health of a society is a non-political issue.

Among the requirements for the implementation of paragraph 7 of the general health policies is “creating the post of deputy or permanent secretary general of health” as a non-political advisory arm for the minister of health due to the non-political nature of the decision-making body in the health system.

### Inter-sectoral leadership

The findings related to this field indicate the theoretical deficiency of community participation for health and the deficiency of a coherent structural network of intersectoral health stakeholders as the most important challenge.

#### Theoretical deficiency of community participation for health

The first challenge that was mentioned was “the dominance of the technological approach over the social approach to health” which, according to some experts, resulted in “the dimming of the role of the approach of social factors affecting health in some macro-decisions” (P32, P29). Also, one of the principles of a good governance system is the attention paid to the “principle of people’s participation in the administration of health systems”. Unfortunately, in the country’s health system, the use of people was only in the construction of charitable service units, while the issue of people’s participation in the administration of the country’s health system is not limited to just this issue. Currently, there is no official guarantee for public participation in health in the country; one of the members of the Health and Treatment Commission commented on this matter, *“New laws must be passed in order to encourage people to participate more. I think we still have many legal gaps in this matter.”* (P34). One way to encourage governance participation is to “put the issue of people’s participation in a clear legal framework”.

#### The deficiency of a coherent structural network of intersectoral health stakeholders

One of the commentators mentioned “the smallness of the health system and the largeness of the health sector” and commented as follows: *“Now all successful countries are moving towards increasing the size of the health system and reducing the size of the health sector, so that the borders of the health system and the health sector become less important because the issue of improving health indicators is a public duty by all departments”* (P29). In recent years, appropriate structural efforts, including the formation of the Secretariat of the Supreme Council of Health and Food Safety, have been made in the country, but the current structure has challenges. One of the experts believed that there should be changes in the structure of the members of the province’s health and food safety working group: *“In the structure of the provincial health council, some key institutions are empty”* (P26). Therefore, the revision of the structural framework will strengthen the interdepartmental role of this secretariat at the provincial level as well. Some experts also pointed to the challenge of “low guarantee of implementation of the approvals of the Supreme Council of Health and Food Safety” and did not consider the role of this institution to be very strong in the implementation of intersectoral supervision in the health system. One of the experts commented as follows: *“In the country as a whole, what guarantee is there in the approvals of the Supreme Council of Health and Food Safety? The Ministry of Health, like other executive bodies, is under the supervision of the President, and in the end, their warning cannot be used to punish the government body that violates health regulations. This has a bias and a solution should be provided for the intervention of the judiciary and with the supervision of the Islamic Council in this field”* (P14).

In this regard, it was suggested that the approval process be carried out by an external body such as the president or the first vice president. Nevertheless, from another point of view, one of the interviewees, referring to the new approaches to governance, agrees with the “institutional approach” by creating specific structures to strengthen the role of stewardship and leadership, and the so-called “understanding reductionism and using the network approach instead of the institutional approach”.

Among the proposed requirements were “Establishment of an organization to protect the rights of health service recipients”, “Development and implementation of the supervision development program of city and village Islamic councils for the provision of health services”, “Designing interdepartmental councils outside the Ministry of Health such as the Healthy Food Council, the Clean Air Council, and the Healthy Industries Council”, “Returning welfare to the Ministry of Health”, and “Strengthening the role of national, provincial and local health assemblies”.

Challenges and organizational-operational requirements of the implementation of paragraph 7 of general health policies in the stewardship function are presented in Table 1.

**Table 1:** Challenges and institutional-operational requirements of the establishment of paragraph 7 of general health policies in the function of stewardship

**Function**	**Sub function**	**Operational Institutional challenge**	**Sub topic**	**Operational Institutional requirements**
**Stewardship**	Evidence informed policy making	Inappropriate Structure	• Defects in adhering to the plan and not being forward-looking of the ministry • lack of a powerful trustee • Dispersion of evidence production institutions • Non-integrated stewardship in specialized departments • Weakness of the institutional position of the Academy of Medical Sciences	• Creating an information and evidence management center under the sub-category of the deputy director of policy, planning and supervision • Establishing Iran’s health statistics, information and evidence organization
		Uncoordinated Processes of Producing Evidence	• Different interpretations of the broad terms of policy making in the health system • Weakness of ideological discussions • politicization • Lack of a network of policymaking experts • The ambiguity in the prioritization of the written policies • Inefficiency in the performance management process • Lack of strong connection between the academic field and implementation	• The formation of a coherent and coordinated network consisting of executive policy makers and university academic professors • The integration of the country’s health system through the coordination of institutions based on the context, content and service process with the responsibility and centrality of the Ministry of Health
		Improper Decision-Making and Management Style	• Insufficient use of tested evidence • Short decision-making life of policy makers and managers • Pressing the levers of power to make policies like some MPs • Separation between policy-making and strategic planning	• Designing a comprehensive system of macro-health policy making • Revitalizing the former health policy making council
		Insufficient Resources	• Policy makers’ insufficient time due to their busy schedule • Lack of trust in policy-making graduates • Inadequate IT infrastructures in the field of decision-making • Insufficient use of experienced and expert advisors	• Creating a health think tank by taking advantage of the potential of the private sector
	**Internal Governance**	Weakness of Governance in Implementation	• Lack of serious commitment and determination • Ambiguity in the executive guarantee of paragraph 7 of general health policies • Severe conflict of interests at different institutional levels • Factional political competition at the decision-making levels in different layers of the health system • The non-implementation of the framework for the establishment of general health policies	• Drafting a specific policy legal framework
		The Weakness of the Role of Regulatory Institutions	• the passive role of the Boards of Trustees of Headquarters and Universities • Insufficient independence of universities in decision-making • Fragmentation in the regulatory system • Ambiguity in the role of external institutions’ supervision	• Strengthening the capacity of the secretariat of the Board of Trustees and the use of control levers, especially budget and financial control tools • Approving and correcting the Law of Independence of Universities • compilation of governance guidelines, issuance of licenses, certification, and accreditation • establishing a deputy for policy, planning and supervision • setting up an independent supervisory unit at the province and city level
		Too Much Tenure of the Headquarters	• Uncertainty in the limits and size of the government in governance and tenure duties • Excessive dependence of environmental units on headquarters due to conflict of interests • Non-separation of functions • Scattering of powers	• Reducing the density of executive tasks of the headquarters and delegating authority to peripheral units • Organizing universities of medical sciences in the form of ten macro-regions, delegating headquarters’ powers to macro-regions and universities
		Incorrect architecture of the structure of headquarters and universities	• Political discontinuity and lack of integrity in stewardship • Parallel work and incorrect definition of roles • Designing a miniature and mirror structure for the headquarters and universities • Not complying with the principles of horizontal, vertical and geographic separation • Transcendent factors to create/remove an organization or structure	• Revitalization of provincial health organizations • Creation of independent and semi-independent units • Reduction of the General Administration in each deputy to a maximum of 3 to 4 General Administrations • Organizing universities of medical sciences in the form of ten macro-regions, delegating headquarters authority to macro-regions and universities
		Contradictory Laws with General Health Policies	• Invalid and cumbersome laws and regulations in the health system • Legal chaos of the health system • Low enforcement guarantee of upstream laws	• Legislative approval in line with the implementation of paragraph 7 by the regulatory institutions • Revision of Invalid Laws and Regulations of the Health • Revision of the Organization Law of the Ministry of Health • Approval of laws such as transparency in the health system and conflict of interest in the health system
		Weakness in the System of Appointing and Dismissing Managers	• Weakness in the regulations for the appointment of managers • Non-separation of technical and political positions	• creating the post of deputy or permanent secretary general of health • Professionalism and strengthening the supervisory position of the ministry in the selection and appointment of managers through the establishment of a center for evaluating the competence of managers
	**Inter-Sectoral Leadership**	Theoretical Deficiency of Community Participation for Health	• The dominance of the technological approach over the social approach to health • Failure to pay attention to the principle of people’s participation in the management of health systems • Weakness in the legal and political frameworks of participation	• Placing the issue of people’s participation in a clear legal framework • Greater participation of municipalities in the administration of health related affairs by the people
		The Deficiency of a Coherent Structural Network of Inter Sectoral Health Stakeholders	• The smallness of the health system and the largeness of the health sector • Low guarantee of implementation of the approvals of the Supreme Council of Health and Food Safety • The governance of the institutional approach	• Establishment of an organization to protect the rights of health service recipients • Development and implementation of the supervision development program of city and village Islamic councils for the provision of health services • Designing interdepartmental councils outside the Ministry of Health: such as the Healthy Food Council, the Clean Air Council, and the Healthy Industries Council • Returning welfare to the Ministry of Health • Strengthening the role of national, provincial and local health assemblies

## Discussion

In the present study, the opinions of experts and authorities from different institutions and organizations, as well as those with different scientific and practical backgrounds, were analyzed regarding Challenges and Executive Requirements of Advanced Health System Governance Based on General Health Policies.

In the context of issues in the field of evidence informed policy making, findings showed that the inconsistent structure and process, especially in the context of the Ministry of Health not being forward-looking, the lack of prioritization of policies and programs, the lack of integrated stewardship, and the lack of communication between different areas are among the existing challenges. The multiplicity of stewardship and supervision ultimately leads to the occurrence of friction between programs and executive processes [32]. Thomas et al. (2020) conducted a study with the aim of strengthening the resilience of health systems and pointed out that organizational structures in the health field, effective information flows in this field, increasing flexibility or the appropriate response capacity, and the integrity of stewardship and supervision in the healthcare system can reduce the challenges of the health system policy field [33].

The existing challenges in the field of policy formulation and decision-making included the dispersion of evidence production institutions, the absence of a single trustee, and the lack of constructive communication. Furthermore, the change in macro-policies and the lack of benefiting from sound scientific evidence in decision-making were other challenges in this field. Finally, from the point of view of the experts, insufficient resources and inappropriate use of policy experts and expert consultants, along with insufficient consensus on policy concepts, have challenged policy formulation and decision-making. Meanwhile, the integration and integrated management of evidence production institutions is recommended. In this regard, Petrescu et al. (2021) in a study emphasized the necessity of integrated management systems in order to improve performance in a dynamic environment with many challenges and opportunities. The integrated management of evidence production institutions is essential for quality management, improving internal communication, reducing costs, and increasing the efficiency of activities through reducing time, bureaucracy and better utilization [34]. In Behzadi Far et al.’s study (2022), the role of evidence-based research in policy-making has been repeatedly emphasized, but the use of this research in decision-making has been limited [35]. Patrício et al. (2020) conducted a study with the aim of designing services for the transformation of health care towards people-centered, integrated and technology-based health care systems, and on the integration of important issues in the field of health and treatment and the lack of fragmentation of the organizational structure, they pointed out and also emphasized that designing services in an integrated manner can help the evolution of health service systems [36], which is consistent with the findings of the present study. In the studies by Conallin et al. (2022) and Al-Ubaydli et al. (2021), the important role of evidence-based research in policymaking has been emphasized a lot [37, 38].

The proposed requirements in the field of evidence informed policy making included the formation of a coherent and coordinated network consisting of executive policymakers and the integration of the country’s health system through the coordination of institutions based on the context, content and process of service with the responsibility and focus of the Ministry of Health. In this regard, Christensen (2021) as well as Androutsopoulou et al. (2018) emphasized the necessity of creating mechanisms for dialogue and consensus among experts in policy-making, and from their point of view, success in this matter should be achieved through a micro-collective path [39, 40].

Some of the problems in the field of intra-departmental governance included the lack of commitment, determination and will, the lack of executive guarantee in paragraph 7 of the general policies, and the conflicts of interest among managers and officials, challenging the reforms to strengthen this sector. The supportive governance environment, political will and consensus of opinions, support by official laws, support resources, coordination and communication, and correct orientation are the most important components introduced by Hsu et al. (2020) in order to facilitate success in the field of health system reforms [41]. In a qualitative study, Masefield et al. (2020) investigated the challenges of effective governance in a low-income health care system and concluded that resolving conflicts of interest can reduce health governance problems and lead to success in low-income health care systems [42], which is consistent with the results of the present study. In Tosanloo et al.’s study (2022), it was also pointed out that solving organizational conflicts can increase the quality of decision-making and, in turn, improve the effectiveness of the organization [43].

The weakness of regulatory and supervisory institutions, including the passive role of university trustees, insufficient independence of universities, fragmentation of the supervisory system, and ambiguity in the supervisory role of external institutions are other problems in the field of governance. In the observation report of the National Institute of Health Research, Mahdavi et al. (2017), besides introducing these challenges and also other challenges in the field of governance, attribute the source of these challenges and problems to non-compliance with upstream documents, lack of enforcement guarantees for laws, and also widespread conflicts of interest in the management domain of the Ministry of Health [44]. Other issues in this field are related to the affluence of the ministry body and excessive management, lack of separation of functions and dispersion of authorities, and lack of integrity of the stewardship. Sometimes the multiplicity and inconsistency of laws has caused disorder in this field and weakened the executive guarantee of upstream documents. The lack of capacity in the Ministry of Health for governance is often proposed as an explanation for the failure of health systems in low- and middle-income countries [12]. Governance affects other functions of the health system, and good governance plays an important role in improving system performance and ultimately health outcomes [45]. In a study, Debie et al. (2022) investigated the successes and challenges of health system governance in the direction of universal health coverage and global health security. Decentralization of healthcare services to public levels, support for beneficiaries, equitable participation and distribution of resources are necessary to support the implementation of programs towards effective governance and security in the healthcare sector. It is also vital to ensure the independent regulatory accreditation of organizations in the health system and the integration of health service indicators related to quality and equality in the national social support supervision and assessment system. Therefore, the existence of effective supervision systems is one of the main components of success in healthcare system policymaking [46].

The requirements of the intra-departmental governance included the creation of a deputyship for policy, planning and supervision, the creation of a provincial supervision unit, the establishment of an independent supervision unit at the province and city level, the formulation of governance guidelines, the issuance of licenses, certificates, accreditation and renewal, and the approval and correction of the University Independence Law. Creating the position of deputy or permanent general secretary of health as a non-political advisory arm for the minister of health and the establishment of a center for assessing the competence of managers were among other requirements. In a study, Mosadeghrad et al. (2011) have presented the challenges of the governance model of Iran’s health system. The findings of this study showed that in the field of governance, the multiplicity of involved institutions has reduced the dynamics and constructive communication, and therefore, by consolidating related functions under specialized units, it can be expected that the governance and supervision dimension will be strengthened [25].

Improper organization of communication mechanisms and stakeholder engagement in the health sector is one of the challenges of intersectoral leadership, which in this field can be attributed to a high tendency towards technology and neglect of the social approach, the smallness of the health system and the size of the health sector, the non-compliance of the stakeholders to the approvals of the Supreme Council of Health and Food Security, and the rule of institutional approaches. Furthermore, to improve intersectoral leadership, people’s participation in this field should be considered. Also, the requirements such as the creation of a support organization for health service recipients, the formulation and implementation of the development plan for the supervision of city and village Islamic councils on the provision of health services, the design of interdepartmental councils outside the Ministry of Health such as the Healthy Food Council, the Clean Air Council and the Healthy Industries Council, and the return of the Welfare Organization to the Ministry of Health have been mentioned. There are various pieces of evidence that have addressed the importance of intersectoral leadership and ways to strengthen it. In Alikhani et al.’s study (2020), it is recommended to focus on the external factors of the health system, especially on the role of the government in creating coordination and support [47]. Moreover, focusing on cross-sectoral coordination as one of the requirements of governance for health has been suggested by Vahadani-nia et al. (2018) [48]. In external evidence, the necessity of comprehensive dialogues with the presence of all stakeholders, attracting the participation of public institutions, strengthening public participation through the creation of independent commissions, and the creation of the National Health Council as the main authority for inter-sectoral leadership has been emphasized [49, 50].

Sustainable development goals have also emphasized the need for the Ministry of Health to involve several sectors towards common goals [51]. The Ministry of Health must negotiate with other government departments, meet and collaborate with a range of non-governmental players at national and international levels, and manage regulatory relationships with stakeholders such as businesses or the health industry [52]. As the needs of populations and health systems are constantly evolving, it is essential to integrate health innovations into such systems in order to provide solutions to existing and emerging needs [53].

According to WHO, “Health innovation identifies new or improved health policies, systems, products and technologies, and services and delivery methods that improve people’s health and well-being.” Stakeholder analysis may be defined as an approach that finds out who the stakeholders are in the health innovation planning process and, based on information gathered about them (e.g., characteristics and relationships), prioritizes who should be involved or have been involved in such planning. Conducting a stakeholder analysis may help: (1) understand the context in which the innovation is developed and implemented; (2) inform the planning process of individuals, groups, or organizations to be involved; and (3) develop strategies to support the development and proper implementation of innovation and prevent possible barriers to its integration [54], which is consistent with the findings of the present study regarding the role of health sector stakeholders in health governance.

### Limitation

This research was constrained by the inconsistency between stakeholders’ expressed positions and their inferred actual interests. Furthermore, the confidential nature of some data limited the scope of the analysis. To address these limitations, a triangulation approach was employed, comparing stakeholders’ stated positions with their actions and the perceptions of other stakeholders. Email correspondence was used to triangulate data and resolve ambiguities.

## Conclusion

The findings of this study can be provided as a rich source of information to policymakers and managers in the field of health and treatment and can be a guide to overcome the current situation in order to realize and establish paragraph 7 of the general policies of the health system. This evidence can create a simplistic attitude in policymakers, and by facilitating evidence-informed policymaking, it can provide the basis for improving the effectiveness of the system. The most important challenges in the field of stewardship include fragmentation, lack of integrated information system and coherent and coordinated evidence production institutions, and weakness in the supervisory dimension due to the affluence of the structure, conflict of interests, and lack of transparency in responsibilities and roles.

It appears to be effective in the establishment of comprehensive stewardship frameworks, the development of an information management system, the coordination of institutions responsible for evidence production, the enhancement of capacity to fortify intra-sectoral governance and inter-sectoral leadership, as well as the creation of an appropriate platform for public engagement, all aimed at addressing existing limitations and challenges. Furthermore, it is recommended that effective legislation be formulated and enacted to promote transparency and mitigate conflicts of interest within the health sector. The integration of information systems, with the objective of institutionalizing the principle of evidence-informed policymaking and achieving integrated stewardship, constitutes another significant proposal aligned with the realization and implementation of paragraph 7 of the general health system policies.

## Supplementary Information

Supplementary Material 1.

Supplementary Material 2.

## Data Availability

No datasets were generated or analysed during the current study.

## Abbreviations

WHO: World Health Organization
UHC: Universal Health Coverage
MOHME: Ministry of Health and Medical Education
PHC: Primary Health Care
HRH: Human Resources for Health
OOP: Out-Of-Pocket
IHIO: Iranian Health Insurance Organization
SSO: Social Security Organization
MOCWSL: Mission and Vision—Ministry of Cooperatives, Labor and Social Welfare
S7GHP: Section 7 of General Health Policies
MP: Members of Parliament
