# Supplementary material for: Challenges and executive requirements of advanced health system governance based on general health policies in Iran: qualitative research
Source: BMC Health Serv Res. 2024 Nov 30;24:1517. doi: 10.1186/s12913-024-11887-z (PMC11608473; doi:10.1186/s12913-024-11887-z)
Supplement: Supplementary file 1 — Supplementary Material 1. [file 12913_2024_11887_MOESM1_ESM.docx]

**Appenix1: Characteristics of qualitative interview participants.**

| **Sex** | **Age** | **length of experience** | **Code** | **Interest of area** | **Organization** | **Specialist** | **Row** |
| --- | --- | --- | --- | --- | --- | --- | --- |
| Male | 52 | 24 | P3 | Stewardship | Ministry (ministerial area, Vice President of Development, Vice President of Health, Vice President of Treatment, National Research Institute, Secretariat of the Supreme Council of Health and Food Security, etc.) | MD | 1 |
| Male | 52 | 26 | P10 | Financing& Stewardship |  | MD-Ph.D | 2 |
| Male | 61 | 25 | P31 | Stewardship |  | Specialist physician | 3 |
| Male | 41 | 10 | P9 | Stewardship |  | MD | 4 |
| Male | 53 | 23 | P7 | Service Delivery & Stewardship |  | Specialist physician | 5 |
| Male | 48 | 20 | P6 | Stewardship |  | PhD in health policy | 6 |
| Male | 51 | 23 | P11 | Financing& Stewardship |  | MD-Ph.D | 7 |
| Male | 51 | 23 | P37 | Stewardship |  | MD-Ph.D | 8 |
| Male | 47 | 19 | P4 | Stewardship |  | MD-Ph.D | 9 |
| Female | 81 | 47 | P22 | Stewardship |  | PhD in health management | 10 |
| Male | 48 | 18 | P29 | Stewardship |  | Specialist physician | 11 |
| Male | 42 | 12 | P18 | Stewardship |  | PhD in health policy | 12 |
| Male | 54 | 27 | P12 | Financing& Stewardship |  | MD-Ph.D | 13 |
| Male | 61 | 32 | P24 | Stewardship |  | PhD in health management | 14 |
| Male | 66 | 38 | P32 | Service Delivery & Stewardship |  | Specialist physician | 15 |
| Male | 47 | 20 | P21 | Stewardship |  | PhD in health policy | 16 |
| Male | 55 | 25 | P30 | Service Delivery |  | PhD in health management | 17 |
| Male | 53 | 23 | P27 | Financing | Health Insurance Organization | Specialist physician | 18 |
| Male | 62 | 29 | P35 | Financing |  | Specialist physician | 19 |
| Female | 33 | 6 | P28 | Financing |  | PhD in health policy | 20 |
| Male | 47 | 22 | P1 | Financing |  | MD-Ph.D | 21 |
| Male | 39 | 11 | P8 | Financing | Social Security Organization and Armed Forces Medical Service Organization | PhD in health management | 22 |
| Male | 51 | 21 | P2 | Financing |  | MD | 23 |
| Male | 52 | 24 | P40 | Financing |  | MD | 24 |
| Male | 81 | 43 | P45 | Stewardship | Medical Sciences Academy | Specialist physician | 25 |
| Male | 79 | 41 | P44 | Stewardship |  | Specialist physician | 26 |
| Male | 81 | 43 | P46 | Financing |  | Specialist physician | 27 |
| Male | 53 | 23 | P19 | Stewardship | Vice-Chancellors of Medical Sciences University | Specialist physician | 28 |
| Male | 50 | 18 | P5 | Stewardship |  | Specialist physician | 29 |
| Male | 56 | 26 | P17 | Stewardship | Parliament | MD-Ph.D | 30 |
| Male | 54 | 24 | P34 | Financing |  | Specialist physician | 31 |
| Male | 46 | 18 | P38 | Stewardship |  | PhD in health management | 32 |
| Male | 64 | 35 | P41 | Stewardship |  | Specialist physician | 33 |
| Male | 69 | 40 | P43 | Financing& Stewardship |  | Specialist physician | 34 |
| Male | 51 | 21 | P13 | Stewardship | Other organizations (Employment Office, Program and Budget, Medical System) | PhD in health policy | 35 |
| Male | 55 | 22 | P36 | Service Delivery |  | Specialist physician | 36 |
| Male | 48 | 21 | P42 | Stewardship |  | MD-Ph.D | 37 |
| Female | 41 | 10 | P15 | Stewardship | Expert | PhD in health policy | 38 |
| Male | 49 | 20 | P39 | Service Delivery |  | MD-Ph.D | 39 |
| Male | 55 | 27 | P26 | Financing& Stewardship |  | MD | 40 |
| Male | 44 | 13 | P14 | Stewardship |  | PhD in health management | 41 |
| Male | 40 | 11 | P23 | Stewardship |  | MD-Ph.D | 42 |
| Male | 48 | 19 | P16 | Stewardship |  | MD-Ph.D | 43 |
| Male | 52 | 26 | P33 | Service Delivery & Stewardship |  | PhD in health management | 44 |
| Male | 62 | 34 | P20 | Service Delivery & Stewardship |  | MD-Ph.D | 45 |
| Male | 44 | 16 | P25 | Stewardship |  | PhD in health policy | 46 |
| Male | 48 | 17 | P47 | Stewardship |  | MD-Ph.D | 47 |
