# Supplementary material for: Challenges and executive requirements of advanced health system governance based on general health policies in Iran: qualitative research
Source: BMC Health Serv Res. 2024 Nov 30;24:1517. doi: 10.1186/s12913-024-11887-z (PMC11608473; doi:10.1186/s12913-024-11887-z)
Supplement: Supplementary file 2 — Supplementary Material 2. [file 12913_2024_11887_MOESM2_ESM.docx]

**Appendix 2: Interview Guide**

Dear Professor:

Greetings and respect

I am in the stage of writing my thesis **"Survey status of operational institutional of health System functions based on General health policies and recommend policy options "**. As you have recalled, paragraph 7 of the general health policies and article 72 of the Sixth Five-Year Development Plan (2016-2021) has determined the main framework for redesigning the functions of the country's health system.

Therefore in this interview, we intend to use your knowledge and experience in order to fulfill the clause 7 general health policies in the form of the following questions. Therefore, please help me in the implementation of this research by participating in the interview.

Thank you in advance for your assistance.

**Paragraph 7 of general health policies**

Separation of the duties of stewardship, financing and Health service provision with the aim of accountability, fulfillment of justice and providing optimal medical services to the people as follows:

**7-1:** Health system stewardship including executive policies, strategic planning, evaluation and monitoring by Ministry of Health and Medical Education

**7-2:** Management of health resources through the insurance system centered on the Ministry of Health and Medical Education and the cooperation of other centers and institutions.

**7-3:** Provision of services by service providers in public, public-private and private sectors.

**7-4:** The coordination and organization of the above matters is according to the mechanism that will be determined by the law.

**A: General question about paragraph 1 of general health policies**

1- Please introduce yourself briefly and give a summary of your executive experience and expertise.

2- Has the policy for the reform of the macro structure of health reached its goals so far? Why Finding Your “Why?” Is Important?

3- What is your opinion about the concept of separation of duties of stewardship, financing, service provision? Do you agree with this distinction? If the answer is yes, please mention, in your view, what was the policymaker's primary goal of separation of duties?

4- In your opinion: - According to paragraph 7-1

- What are the challenges in the field of policy-making and evidence generation in Iran's health system?
- What challenges are there in the field of Iran's intra-sectoral governance?
- What challenges are there in the field of Iran's intersectoral leadership dimension?
- In your opinion, what challenge in the health system does the policymaker mean by strategic planning, executive policy making, and monitoring and evaluation?
- In order to solve these challenges, what requirements do you suggest in general?

5- General health policies: - According to paragraph 7-2

- In your opinion, what does the policymaker mean by the centrality of the Ministry of Health in resource management?
- What is meant by the cooperation of other centers and institutions?
- In your opinion, what does the policymaker mean by the insurance system in this paragraph?
- What are the current challenges in this field in the country's health system?
- In order to solve these challenges, what requirements do you generally suggest?

6- In your opinion, what are the challenges in the way of the health system to fulfill paragraph 7-3 and what requirements do you suggest to solve them?

7- What is your executive proposal (not theoretical and ideal) to maintain the position of the Ministry of Health and, perform the task of separating service delivery, stewardship and financing?

**B: Role players and beneficiaries (position, power and interest)**

In your opinion, briefly, what is the role of each of the following bodies in the implementation of paragraph 7-1 of general health policies?

8- Which institutions are the main players in order to draw and better implement this policy?

9- From your point of view, in the organization where you serve, what criteria and principles should exist for the establishment of paragraph 7 of general health policies?

10- How do you evaluate your organization from the point of view of position, power and interest to establish the provisions of paragraph 7 of general health policies? Do you think there is a specific institution/association or organization that is very influential on the establishment of paragraph 7 of general health policies?

11- What strategy and strategies do you suggest in order to interact with the stakeholders?

12- If it is necessary to mention another point here that has been omitted from my questions, please mention it?
